# Conservation of *shibire* and *RpII215* temperature-sensitive lethal mutations between *Drosophila* and *Bactrocera tryoni*

**DOI:** 10.3389/finsc.2024.1249103

**Published:** 2024-03-04

**Authors:** Thu N. M. Nguyen, Amanda Choo, Simon W. Baxter

**Affiliations:** ^1^ School of BioSciences, University of Melbourne, Melbourne, VIC, Australia; ^2^ School of Biological Sciences, University of Adelaide, Adelaide, SA, Australia

**Keywords:** temperature sensitivity, embryo lethality, transgenic complementation test, *shibire*, *RNA polymerase II 215*

## Abstract

The sterile insect technique can suppress and eliminate population outbreaks of the Australian horticultural pest, *Bactrocera tryoni*, the Queensland fruit fly. Sterile males mate with wild females that produce inviable embryos, causing population suppression or elimination. Current sterile insect releases are mixed sex, as the efficient removal of unrequired factory-reared females is not yet possible. In this paper, we assessed the known *Drosophila melanogaster* temperature-sensitive embryonic lethal alleles *shibire* (G268D, *shi^ts1^*) and *RNA polymerase II 215* (R977C, *RpII215^ts^*) for potential use in developing *B. tryoni* genetic sexing strains (GSS) for the conditional removal of females. Complementation tests in *D. melanogaster* wild-type or temperature-sensitive genetic backgrounds were performed using the GAL4–UAS transgene expression system. A *B. tryoni* wild-type *shibire* isoform partially rescued *Drosophila* temperature lethality at 29°C by improving survivorship to pupation, while expressing *B. tryoni shi^ts1^* failed to rescue the lethality, supporting a temperature-sensitive phenotype. Expression of the *B. tryoni RpII215* wild-type protein rescued the lethality of *D. melanogaster RpII215^ts^* flies at 29°C. Overexpressing the *B. tryoni RpII215^ts^* allele in the *D. melanogaster* wild-type background unexpectedly produced a dominant lethal phenotype at 29°C. The *B. tryoni shibire* and *RpII215* wild-type alleles were able to compensate, to varying degrees, for the function of the *D. melanogaster* temperature-sensitive proteins, supporting functional conservation across species. *Shibire* and *RpII215* hold potential for developing insect strains that can selectively kill using elevated temperatures; however, alleles with milder effects than *shi^ts1^* will need to be considered.

## 1 Introduction

Insect pests pose a constant and significant threat to the agriculture sector and animal livestock industries, as they can cause serious negative impacts on productivity, impose market access restrictions, and threaten food security. Integrated pest management strategies aim to keep insect pest populations below levels that cause economic loss by combining environmentally acceptable control methods and sustainable farming techniques (1–3). These strategies help reduce the use of insecticides, which often have adverse effects on non-target animals and result in the development of insecticide resistance in pest populations (4, 5). The sterile insect technique (SIT) is an efficient species-specific insect biocontrol strategy that is used as a component of integrated pest management (3). SIT involves the intentional release of large numbers of sterile insects into regions with low to moderate pest density to decrease or eliminate pest populations (6). Radiation exposure to factory-reared pupae causes irreparable chromosomal damage and sterility. Once development is complete, the sterile insects are released, and males that mate with wild females produce inviable embryos, causing reproductive failure and population suppression. SIT was first developed to control New World screwworm (7) and has since successfully been used to combat numerous pest species worldwide, including several species of tephritid fruit flies (8), moths (9), tsetse flies (10), and mosquitoes (10, 11).

The Queensland fruit fly, *Bactrocera tryoni* (Diptera: Tephritidae), can infest more than 200 fruits and vegetables and is the most significant pest threat to Australian horticultural industries (12). Females oviposit into soft and ripening host fruit, resulting in puncture wounds that are susceptible to microbial infection, and larvae feed on internal flesh, causing severe damage (12). Controlling *B. tryoni* has traditionally relied on trapping systems containing attractants or broad-spectrum insecticides that have posed environmental and human health concerns (13). SIT has been applied in the management of *B. tryoni* to restrict reproduction and eliminate outbreaks (14). The current SIT approach for *B. tryoni* releases both sterile males and females, although females are not required. Developing strategies for sterile male-only release will significantly enhance the efficacy and cost-effectiveness of the program, which has been proven in another tephritid species, the Mediterranean fruit fly (Medfly), *Ceratitis capitata* (15).

Medfly SIT programs have benefited from genetic sexing strains (GSS) that enable male-only releases. GSS females are homozygous for a *temperature-sensitive lethal* (*tsl*) mutation causing embryonic death when treated at 34°C for 24 h, whereas males survive heat treatment as they carry a wild-type chromosomal genetic region translocated to the Y chromosome (16, 17). The Medfly GSS has taken decades to develop (8), and the *tsl* was generated by random mutagenesis, with its genetic nature remaining unknown (18). This female conditional lethal strategy has the potential to be replicated in other tephritid species using precise CRIPSR/Cas9 genome editing technology (19); however, this would require identification of the Medfly *tsl* mutation and its ortholog in the target species. An alternative strategy is the utilization of known temperature-sensitive mutations that have been characterized in the vinegar fly, *Drosophila melanogaster* (20–22).

Three key requirements exist for temperature-sensitive mutations to be considered as potential candidates for the development of an ideal GSS: i) Lethality should occur at early developmental stages, when embryos are treated at a restrictive temperature; ii) homozygous mutant females must be viable and fecund at permissive rearing temperatures for large-scale production; and iii) the conditional mutation should be recessive so that male fitness can be rescued using a wild-type allele translocated to the Y chromosome. A number of temperature-sensitive mutations have been identified in *D. melanogaster* (20, 23–25), yet only a few of them meet these three important requirements (26), e.g., *shibire* (*shi*) and the *RNA polymerase II 215* (*RpII215*) genes (**Table 1**).

**Table 1:** Characterized *Drosophila melanogaster* temperature-sensitive mutations with potential to cause embryonic lethality when introduced into other insect species.

Name (symbol)	Chromosome location^a^	Mutation	Amino acid substitution	Permissive temperature	Restrictive temperature	Phenotype (restrictive temperature)	References
*shibire* (*shi*)	X 15,892,116 to 15,906,716 [+]	*shi^ts1^*	G268D	22°C	27–29°C	Embryonic lethal	(20, 26, 27)
		*shi^ts2^*	G141S	22°C	27–29°C	Semi-embryonic lethal	
*RNA pol. II 215kD subunit* (*RpII215*)	X 11,562,800 to 11,570,326 [−]	*RpII215^ts^*	R977C	22°C	29°C	Embryonic or early larval lethality^b^	(28)

Table was adapted from Nguyen, Choo (26).

[+] sense DNA strand, [−] anti-sense DNA strand.

a NCBI reference sequences for Drosophila melanogaster X are NC_004354.4.

b Complete lethality is observed when embryos are incubated for >9 h at 29°C.

Using the mutagen ethyl methanesulfonate (EMS), Grigliatti et al. (20) isolated six temperature-sensitive mutations in the *D. melanogaster* X-linked *shibire* locus (*shi^ts1^* to *shi^ts6^*), which encodes a GTPase dynamin protein involved in synaptic vesicle recycling in nerve terminals (29). Four of these mutations (*shi^ts1^*, *shi^ts2^*, *shi^ts3^*, and *shi^ts6^*) had been reported to have partial or complete embryonic lethality at 29°C and are unaffected when reared at 22°C (20). The *D. melanogaster shi^ts1^* (G268D) and *shi^ts2^* (G141S) alleles are point mutations resulting in amino acid substitutions at the boundary of the *shibire* GTPase domain (30) and also cause paralysis at the larval and adult stages at 29°C (20, 27). The mutations underlying *shi^ts3^* and *shi^ts6^* have never been reported.

Heat treatment of *shi^ts2^* embryos at 29°C results in a less severe phenotype than *shi^ts1^* (26). Individually expressing six different *shibire* wild-type isoforms in a *shi^ts2^* background revealed that specific isoforms were able to rescue some of the temperature-sensitive phenotypes—this includes rescuing the lethality of *shi^ts2^* larvae subjected to heat pulses late in development (at 32°C, 34°C, and some at 36°C) and adult paralysis at 27°C (31). The rescue of adult paralysis was considered partial as the rescue flies were still paralyzed at higher temperatures of 30°C and 32°C, suggesting that a single isoform of the gene may not be sufficient to rescue completely (31).

The X-linked gene *RpII215* encodes for a subunit of RNA polymerase II, a multi-subunit enzyme catalyzing the transcription process to synthesize various types of RNA (32). The *RpII215* temperature-sensitive (*RpII215^ts^*) mutation was induced using EMS by Mortin and Kaufman (28). Initial reports showed that the *RpII215^ts^* mutants were viable at the permissive temperature of 22°C, whereas shifting embryos to a high temperature of 29°C resulted in lethality at the late embryo or first larval instar stages (23, 28). Nguyen et al. (26) found that 25°C is also a suitable permissive temperature for rearing *RpII215^ts^* homozygous mutants. The *RpII215^ts^* mutation is a single-base substitution resulting in an amino acid replacement (R977C) located in domain 6, which forms part of the shelf module (33, 34).

Here, we used the *D. melanogaster* GAL4-UAS system to perform complementation tests by expressing *B. tryoni* transgenes for *shibire* or *RpII215* in *Drosophila* temperature-sensitive genetic backgrounds to determine whether phenotypic rescue could be achieved. We hypothesized that phenotypic rescue would occur at 29°C through the expression of *B. tryoni* wild-type alleles and that synthetic alleles containing *shi^ts1^* (G268D) or *RpII215^ts^* (R977C) substitutions would fail to rescue. This work advances our understanding of temperature-sensitive alleles in *B. tryoni* and provides new candidates for developing GSS for SIT.

## 2 Materials and methods

### 2.1 *Drosophila melanogaster* strains

The fly strains used in this study are listed in **Supplementary Table S1**. All stocks were maintained on standard cornmeal media at 25°C with 65% relative humidity at a 12:12 light/dark cycle unless described otherwise. Crosses investigating *shibire* temperature sensitivity were performed in the *shi^ts2^* (DGRC 106754) background, as attempts to obtain a *D. melanogaster shi^ts1^* stock (DGRC 106278) that survived the shipping process from the Bloomington Drosophila Stock Center (BDSC) or Kyoto Stock Center (DGRC) were unsuccessful.

### 2.2 Cloning of full-length *Bactrocera tryoni shi^+^, shi^ts1^, RpII215^+^*, and *RpII215^ts^* cDNAs into P-element transformation vectors

The *D. melanogaster* shibire (shi) and RNA polymerase II 215 (RpII215) proteins were queried against the *B. tryoni* genome (GCA_000695345.1) using the tBLASTn algorithm in Geneious (v7.1.7) to identify their orthologs. The predicted coding sequence was obtained from a “gff3” genome annotation file (35). The NCBI Conserved Domain Search (CD-search) was used to identify signature motifs in *B. tryoni* orthologs. Codon optimization was performed on the coding sequence using the Integrated DNA Technologies (IDT, Coralville, IA, USA) online Codon Optimization Tool (http://sg.idtdna.com/CodonOpt) to account for *D. melanogaster* codon bias.

A Kozak sequence (CAAAATG) was placed in front of the start codon to facilitate translation initiation, and the stop codon “TAA” was added at the end of the coding sequence. gBlocks for *B. tryoni shi*, *shi^ts1^*, *RpII215^+^*, and *RpII215^ts^* were obtained from IDT as dried oligos (1,000 ng), which were then resuspended in 20 μL of TE (10 mM Tris, pH 7.5–8.0, 1 mM EDTA) to 50 ng/μL stock solutions and stored at −20°C. gBlock fragments were cloned into the *Eco*RI site of the pUAST-attB vector using Gibson Assembly Master Mix (NEB, Ipswich, MA, USA). Cloned products were analyzed using Sanger sequencing to ensure they did not contain non-synonymous mutations. Validated constructs were transformed into *D. melanogaster* by BestGene Inc. (Chino Hills, CA, USA).

### 2.3 Expression of *Bactrocera tryoni* orthologs under *Drosophila melanogaster* temperature-sensitive mutant backgrounds

The prefix *Q* distinguishes between *B. tryoni* (Queensland fruit fly) transgenes and *D. melanogaster* alleles or genotypes. The *UAS-Qshi^+^* and *UAS-Qshi^ts1^* transgenic lines and *da-GAL4* and *nSyb-GAL4* driver lines were crossed into the genetic background of the *D. melanogaster shi^ts2^* strain (DGRC 106754). The *D. melanogaster* transgenic lines carrying *UAS-QRpII215^+^* and *UAS-QRpII215^ts^* and the *da-GAL4* driver line were crossed into the genetic background of the *D. melanogaster RNA polymerase temperature-sensitive* strain *RpII215^ts^* (BDSC 34755) (**Supplementary Tables S2**–**S4**). The presence of UAS transgenes and *D. melanogaster* mutant backgrounds was confirmed by PCR and Sanger sequencing using the primers listed in **Supplementary Table S5**. GAL4 driver expression was verified by crossing the respective line to UAS-mCD8-GFP, then confirming the green fluorescent protein (GFP) expression in the progeny (data not shown).

Crosses were performed using 3- to 6-day-old virgin flies with three males and five females per vial. Egg laying occurred for 24 h at 25°C and the flies were then removed. Vials containing eggs were maintained at either 18°C, 22°C, 25°C, or 29°C until pupal eclosion, and the number of adult flies, either the GAL4 driver or the balancer TM6B, was recorded. The percentage of progeny with the GAL4 driver (non-TM6B) was calculated for each treatment. Data were analyzed using RStudio (v1.2.1335).

## 3 Results

### 3.1 Generating *Drosophila melanogaster* transgenic lines carrying *Bactrocera tryoni shi* or *RpII215* orthologs

We generated *D. melanogaster* transgenic lines carrying the *B. tryoni* wild-type (*Qshi^+^* and *QRpII215^+^*) and putative temperature-sensitive (*Qshi^ts1^* and *QRpII215^ts^*) transgenes. Expression was selectively controlled using the GAL4–UAS system (**Supplementary Figure S1**) (36). Complementation rescue experiments in the *Drosophila* temperature-sensitive lethal strains *shi^ts2^* and *RpII215^ts^* were then attempted with the *B. tryoni* transgenes. Expressing wild-type allelic transgenes was expected to rescue *Drosophila* temperature-sensitive phenotypes, while putative temperature-sensitive alleles were not expected to rescue.

The *B. tryoni shi^ts1^* (G268D) mutation was investigated as *D. melanogaster shi^ts1^* has complete embryonic lethality. The *B. tryoni* ortholog of the *D. melanogaster* “short” isoform (**Supplementary Figure S2**), which has 49 amino acids absent at the carboxy terminal, was chosen for the transgenic complementation test. The *D. melanogaster shibire* “short” isoform previously appeared to have relatively consistent rescue phenotypes when expressed in the *D. melanogaster shi^ts2^* temperature-sensitive background at high temperatures (31). Staples and Ramswami (1999) showed that a single isoform was unable to completely rescue the temperature-sensitive phenotypes across a broad range of restrictive temperatures, although rescue could occur at specific temperatures and a distinguishable level of rescue was still observable with each isoform (31). Insertion of the entire *B. tryoni* genomic region of *shibire* may have enabled alternate splicing of multiple isoforms; however, this was not feasible due to the size of the genomic region (30 kb). The coding sequence of *B. tryoni shi* “short” was obtained from a gff3 genome annotation file provided with the draft genome sequencing project (35). The shibire proteins from *D. melanogaster* and *B. tryoni* share 91% identity, including conserved glycine amino acids at positions 268 and 141, which are encoded by aspartate in *shi^ts1^* (G268D) and serine in *shi^ts2^* (G141S) mutants, respectively.

The *D. melanogaster RpII215* has only one protein isoform (**Supplementary Figure S3**), which was used in a BLAST search against the *B. tryoni* genome (JHQJ00000000.1) to identify *B. tryoni RpII215* on scaffold Btry263 (JHQJ01000312.1). The coding regions of the two alleles share 78% identity and their protein sequences share 93% identity. The amino acid that is mutated in *D. melanogaster RpII215^ts^* (R997C) is conserved in *B. tryoni*.

### 3.2 Assessing the temperature sensitivity of *Bactrocera tryoni shi* transgenes

The *D. melanogaster shi^ts2^* strain was used as a temperature-sensitive lethal genetic background to test for complementation rescue with the *B. tryoni shi^+^* wild-type construct. Complementation tests using the *B. tryoni shi^ts1^* were also performed, but not expected to rescue the temperature-sensitive phenotype. According to modENCODE (implemented in FlyBase r2021_05) (37) and Chen et al. (38), the *D. melanogaster shi* is expressed in all life stages, and its major expression site is the nervous system. We obtained a stock of *shi-GAL4* (BDSC stock ID 42738) with the intention to drive the expression of the UAS *shibire* constructs with the native promoter; however, we were unable to detect the expression of the UAS responder constructs with this specific driver. Consequently, the drivers *da-GAL4* and *nSyb-GAL4* were chosen due to their similar expression patterns in brain tissues according to single-cell transcriptomic analysis of *D. melanogaster* (39).

Interspecific functional complementation tests were carried out by crossing the UAS responder female lines with the male GAL4 driver lines to test the ability of *B. tryoni* transgenes to rescue the *D. melanogaster shi^ts2^* temperature-sensitive effect at 29°C. The crossing strategy was designed with an internal control, meaning that ~50% of progeny would inherit the *GAL4* driver and express the transgene and ~50% would inherit the visible phenotypic marker *TM6B* and not express the transgene.

**Figure 1:**
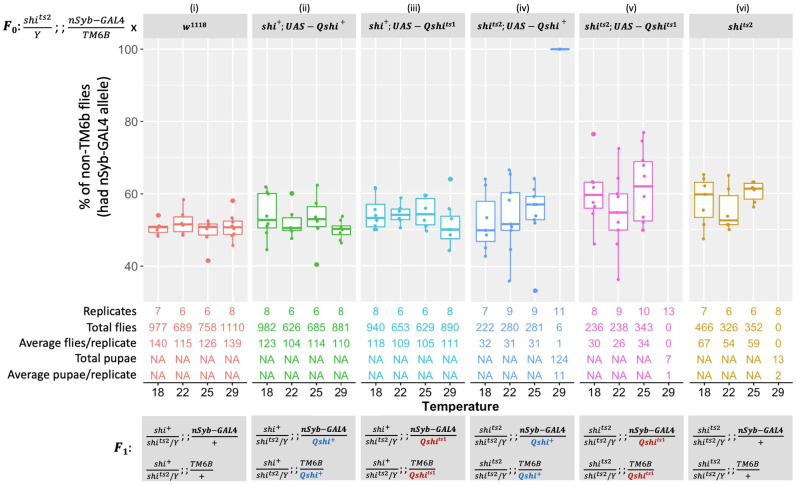
Expressing *Bactrocera tryoni shi* wild-type (*UAS-Qshi^+^*) and mutant (*UAS-Qshi^ts1^*) transgenes driven by the *shi^ts2^; nSyb-GAL4/TM6B* driver. In each standard food vial, F_0_ crosses were set up by placing three males that were hemizygous for the temperature-sensitive *shi^ts2^* mutant allele on the X chromosome, heterozygous for *nSyb-GAL4*, and balancer on the third chromosome (*shi^ts2^/Y; +/+; nSyb-GAL4/TM6B*) and five females of either control wild type (*w^1118^*) (***i***), *Qshi^+^* in the wild-type background (*shi^+^; UAS-Qshi^+^*) (***ii***), *Qshi^ts1^* in the wild-type background (*shi^+^; UAS-Qshi^ts1^*) (***iii***), *Qshi^+^* in the temperature-sensitive background (*shi^ts2^; UAS-Qshi^+^*) (***iv***), *Qshi^ts1^* in the temperature-sensitive background (*shi^ts2^; UAS-Qshi^ts1^*) (***v***), or control temperature-sensitive (*shi^ts2^*) (***vi***). F_0_ parents were allowed to lay eggs for 24 **h** at 25°C, then eggs in vials were reared at either 18°C, 22°C, 25°C, or 29°C. In the F_1_, chromosomes are presented in order: sex determination chromosomes, chromosome 2, and chromosome 3. F_1_ flies with non-TM6B expressed *UAS-Qshi^+^* or *UAS-Qshi^ts1^*, while *TM6B* flies did not express these transgenes. The number of replicates and total counted F_1_ flies for each cross at each temperature were indicated. ***Box plots*** represent the interquartile range, and the median value is indicated. Error bars represent 1.5 times the interquartile range.

Males with the genotype *shi^ts2^/Y; +/+; nSyb-GAL4/TM6B* were systematically crossed with six different homozygous female strains, and progeny were reared at four temperatures (18°C, 22°C, 25°C, or 29°C). Crossing males with *w^1118^* control females confirmed that progeny survived under all experimental conditions and approximately 50% of progeny inherited the TM6B marker [**Figure 1** (i)]. When males were crossed to *shi^ts2^* female homozygotes, strong survivorship was recorded at all temperatures, except at 29°C where only 13 individuals developed to the pupal stage, which is consistent with a previous observation [**Figure 1** (vi)] (20, 26). The *UAS-Qshi^+^* and *UAS-Qshi^ts1^* constructs were then expressed in *D. melanogaster* to assess any negative impacts of expressing the transgenes. Approximately 50% of individuals expressed the transgene at all temperatures, demonstrating that *B. tryoni UAS-Qshi^+^* and *UAS-Qshi^ts1^* do not negatively affect fly viability under the *nSyb-GAL4* driver [**Figure 1** (ii and iii)].

A complementation cross was performed to determine whether the *Drosophila shi^ts2^* temperature sensitivity could be rescued with the *B. tryoni* wild-type *UAS-Qshi^+^* transgene. Flies expressing *UAS-Qshi^+^* under the *nSyb* promoter or lacking expression (TM6B) were recovered in similar proportions at 18°C, 22°C, and 25°C. At 29°C, only six adults expressing *UAS-Qshi^+^* emerged from 11 replicate vials, although there were 124 pupae that died in late-stage development (P12–P14, wings were varied from gray to completely black) (40). In comparison, only 13 pupae from 8 replicate vials were observed in the *shi^ts2^* mutant background [**Figure 1** (vi)]. Expressing the *B. tryoni shibire* “short” isoform, *nSyb-GAL4:UAS-Qshi^+^*, provided partial temperature-sensitive rescue at 29°C in a *Drosophila shi^ts2^* background.

Expression of *UAS-Qshi^ts1^* in the *D. melanogaster* temperature-sensitive *shi^ts2^* background had no adult survival at 29°C. There were seven early-stage pupae that died prior to stage P8, as eyes were not visible [**Figure 1** (vi)] (40). The *B. tryoni shi^ts1^* allele differed in function from *B. tryoni* wild-type *shi^+^* at a high temperature of 29°C [**Figure 1** (v)]. These data indicate that amino acid G268 is required for function at elevated temperatures.

Expression of *UAS-Qshi^ts1^* and *UAS-Qshi^+^* was also assessed using the *da-GAL4* driver. However, dominant lethal effects were observed when *UAS-Qshi^+^* was expressed at 29°C, indicating that the driver was not suitable for the expression of this transgene (**Supplementary Figure S4**).

### 3.3 Assessing the temperature sensitivity of *Bactrocera tryoni RpII215* transgenes

The *D. melanogaster* gene *RpII215* is expressed in all life stages and tissues according to modENCODE (implemented in FlyBase r2021_05) (37). The R977C mutation (*RpII215^ts^*) is temperature-sensitive lethal at 29°C. We hypothesized that expressing *B. tryoni UAS-QRpII215^+^* in temperature-sensitive *Drosophila* (*RpII215^ts^*) would rescue embryonic lethality at 29°C but that *UAS-QRpII215^ts^* would fail to rescue. The *da-GAL4* driver was used as a ubiquitous driver to express UAS constructs, as *RpII215-GAL4* drivers reproducing the complete endogenous expression pattern have not been described.

**Figure 2:**
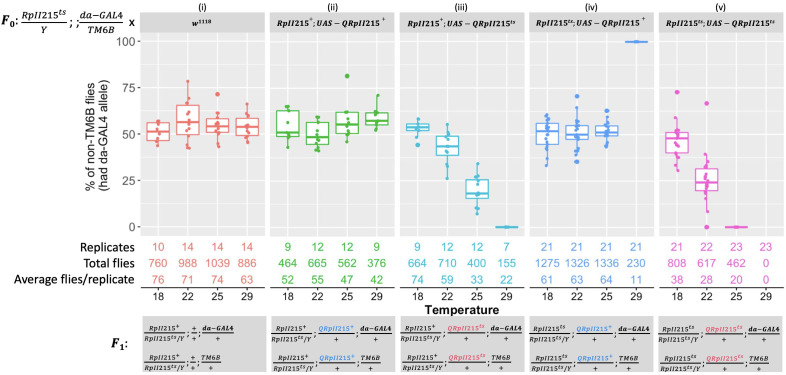
Expressing *Bactrocera tryoni RpII215* wild-type (*UAS-QRpII215^+^*) and mutant (*UAS-QRpII215^ts^*) transgenes with the *da-GAL4* driver. In each food vial, F_0_ crosses were set up by placing three males that were hemizygous for the temperature**-**sensitive *RpII215^ts^* mutant allele on the X chromosome, heterozygous for *da-GAL4*, and balancer on the third chromosome (*RpII215^ts^/Y; +/+; da-GAL4/TM6B*) and five females of either control wild type (*w^1118^*) (*i*), *QRpII215^+^* in the wild-type background (*RpII215^+^; UAS-QRpII215^+^*) (*ii*), *QRpII215^ts^* in the wild-type background (*RpII215^+^; UAS-QRpII215^ts^*) (*iii*), *QRpII215^+^* in the temperature-sensitive background (*RpII215^ts^; UAS-QRpII215^+^*) (*iv*), or *QRpII215^ts^* in the temperature-sensitive background (*RpII215^ts^; UAS-QRpII215^ts^*) (*v*). F_0_ parents were allowed to lay eggs for 24 h at 25°C, then eggs in vials were reared at either 18°C, 22°C, 25°C, or 29°C. In the F_1_, chromosomes are presented in order: sex determination chromosomes, chromosome 2, and chromosome 3. F_1_ flies with non-TM6B expressed *UAS-QRpII215^+^* or *UAS-QRpII215^ts^*, while *TM6B* flies did not express these transgenes. The number of replicates and total counted F_1_ flies for each cross at each temperature were indicated. *Box plots* represent the interquartile range, and the median value is indicated. Error bars represent 1.5 times the interquartile range.

Interspecific functional complementation tests were performed by crossing experimental female lines with the same male driver line. Similar to the crosses described in **Figure 1**, 50% of progeny were expected to carry *da-GAL4* and express the transgene, and 50% should carry TM6B and not express the transgene. Control crosses confirmed that *da-GAL4* expression of the *B. tryoni* wild-type QRpII215^+^ protein did not have adverse effects [**Figure 2** (i and ii)]. Homozygous and hemizygous *RpII215^ts^ Drosophila* only survived at 29°C when the *da-GAL4* allele drove the expression of *UAS-QRpII215^+^*, rescuing lethality and complementing loss of function [**Figure 2** (iv)].

Surprisingly, the expression of the putative temperature-sensitive construct, *UAS-QRpII215^ts^*, showed a dominant lethal phenotype as temperature increased, regardless of genetic background. *Drosophila* carrying a wild-type *RpII215^+^* allele were unable to survive at 29°C [**Figure 2** (iii)]. In the *Drosophila RpII215^ts^* background, flies expressing the *QRpII215^ts^* transgene could not survive at the lower temperature of 25°C and none survived at 29°C [**Figure 2** (v)].

In summary, the *B. tryoni UAS-QRpII215^+^* was able to rescue the lethality associated with *D. melanogaster RpII215^ts^* at 29°C. A dominant negative effect occurred when expressing *UAS-QRpII215^ts^* in *D. melanogaster* with one wild-type allele, as expression of the *RpII215^ts^* itself resulted in reduced viability at 22°C and 25°C and complete lethality at 29°C. A more severe effect was observed with the expression of *B. tryoni UAS-QRpII215^ts^* in the *D. melanogaster* temperature-sensitive *RpII215^ts^* mutant background, which resulted in lethality at 25°C. Amino acid R977 in the *B. tryoni* RpII215 protein was required for viability at certain temperatures.

## 4 Discussion

Despite some success in genetic transformation and genome editing capabilities using molecular technologies, investigating temperature-sensitive lethal mutations in *B. tryoni* remain challenging (41, 42). Generating precise single-base substitutions in *B. tryoni* using CRISPR/Cas9 technology via the homology-directed repair (HDR) pathway is complex (19) and time-consuming, with the potential of off-target effects causing genetic variation. A minimum of three generations is required (which can take up to 3 months) to establish homozygous mutant strains. Therefore, using the *D. melanogaster* GAL4–UAS system can advance functional studies of *B. tryoni* orthologs with the advantage of performing experiments in the same genetic background across all lines in a relatively short time frame. *Drosophila* complementation tests were performed to determine whether *B. tryoni* wild-type alleles could rescue known temperature-sensitive lethal mutations for *Drosophila shibire* and *RpII215*. Substitution of specific amino acids, which are known to cause *Drosophila* temperature sensitivity, into *B. tryoni* alleles was not expected to rescue.

*Shibire* is an essential gene for *D. melanogaster* development as homozygous null mutants are non-viable (43). Multiple isoforms are produced and are likely to be expressed within different tissues at different development stages (31, 38). Staples and Ramaswami (31) reported that temperature-sensitive paralysis in *shi^ts2^* flies occurred at 27°C, and partial phenotypic rescue was achieved through transgenic expression-specific *shibire* isoforms. A series of isoforms described as “short” lacked a 49-amino acid region at the carboxy terminus and prevented paralysis up to 30–32°C. *B. tryoni* is also known to express multiple *shibire* isoforms in RNA-seq transcriptome datasets (Dr. Stephen Pierce, CSIRO, personal communication). Expression of an equivalent “short” isoform of the *B. tryoni* wild-type *shibire* partially rescued the *D. melanogaster shi^ts2^* temperature-sensitive phenotype when expressed using the *nSyb-GAL4* driver. Insertion of the entire *shibire* genomic region would have been ideal to enable the transcription of all isoforms and may have then resulted in improved rescue efficiency; however, plasmid construction and genomic integration were not feasible due the insert size (approximately 30 kbp).

Expression of the *shi^ts1^* mutant allele failed to show any level of rescue of the associated temperature-sensitive lethality, supporting the G268D amino acid substitution as an important site for viability at restrictive temperatures. Choo et al. (19) have successfully introduced this equivalent *shi^ts1^* mutation into the *B. tryoni* genome using CRIPSR/Cas9-mediated homology directed repair. However, the mutation was found to be homozygous lethal at the low rearing temperature of 21°C (19). This shows reduced fitness compared to the equivalent mutation in *Drosophila* (27). The *D. melanogaster shi^ts2^* had been shown to have a milder temperature-sensitive phenotype relative to *shi^ts1^* (20, 27, 44). Although initial reports showed no embryonic lethality in *Drosophila*, Nguyen et al. (26) observed semi-embryonic lethality at 29°C (88% lethality), and no adult survival if continually treated at 29°C. This indicates that *shi^ts2^* can also result in embryonic lethality, but appears less severe than *shi^ts1^*. The *B. tryoni shi^ts2^* could be a potential target for future attempts at creating a GSS.

Dominant negative or semi-dominant effects have been reported for the *Drosophila* UAS-*shi^ts1^* transgene when the construct was intentionally overexpressed at very high levels (45). We did not observe a poisoning effect when the *B. tryoni* UAS-*Qshi^ts1^* was expressed via the *da*-GAL4 or *nSyb*-GAL4 drivers in *Drosophila* wild-type genetic backgrounds at any temperature. A dominant negative effect did occur when the ubiquitous *da*-GAL4 driver (but not *nSyb*-GAL4) expressed the *B. tryoni shibire* wild-type allele at 29°C (**Supplementary Figure S4**ii). The toxic effects appeared to be caused by the specific expression pattern of the *da*-GAL4 driver, which overexpressed the *shibire* “short” isoform in specific tissues. Alternatively, differences in the amino acid sequences between the *D. melanogaster* and *B. tryoni* shibire proteins may have functional consequences in specific tissues.

The *D. melanogaster RpII215* gene encodes for a subunit of a multi-subunit enzyme, RNA polymerase II, involved in transcription catalysis (32). Expression of the *B. tryoni* wild-type *RpII215* was sufficient to rescue the lethality of *D. melanogaster RpII215^ts^* at a high temperature of 29°C, indicating functional conservation across species. The *B. tryoni RpII215^ts^* expressed a dominant lethal effect in the *D. melanogaster* wild-type *RpII215* background resulting in complete lethality at 29°C and had an even more severe effect in the temperature-sensitive *RpII215^ts^* background (when lethality was observed at 25°C). The reasons behind the *B. tryoni RpII215^ts^* temperature-sensitive dominant nature are unclear. Dominant negative effects have been observed expressing other temperature-sensitive alleles. Overexpression of *D. melanogaster shi^ts1^* in different neuronal subsets using the GAL4–UAS system can result in dominant temperature-sensitive effects (41, 46). The *D. melanogaster shi^ts1^* proteins block vesicle endocytosis at high restrictive temperatures (30, 44). In the case of overexpressing *B. tryoni RpII215^ts^* at 29°C, we propose two hypotheses. First, *RpII215^ts^* produces a thermolabile product that is inactivated or degraded at 29°C (23, 42). Alternatively, the assembly of the *RpII215^ts^* subunit into an active RNA polymerase II multi-subunit enzyme was temperature-sensitive (23). The *B. tryoni RpII215^ts^* may or may not have a dominant effect when introduced in the *B. tryoni* genome, which requires further experiments to confirm.

Expressing *B. tryoni RpII215^ts^* in a *D. melanogaster RpII215^ts^* temperature-sensitive lethal background caused complete lethality at 25°C, suggesting that wild-type amino acid R977 in the *B. tryoni RpII215* protein has an important role in viability at certain temperatures. Determining whether the *RpII215^ts^* mutation has a dominant negative effect in *B. tryoni* will require transgenic modification and further experimentation.

## 5 Conclusions

We provide evidence for the partial rescue of *D. melanogaster* temperature-sensitive alleles of *shibire*, and strong rescue for *RpII215*, using interspecific complementation tests with *B. tryoni* wild-type orthologs. These results demonstrate a level of functional conservation across species. The *B. tryoni* equivalent of the temperature-sensitive alleles responded differently compared with the wild-type alleles at specific temperatures, supporting the replacement of amino acids (*shibire* G268D and *RpII215* R977C) that failed to rescue temperature-sensitive phenotypes. *Shibire* and *RpII215* hold some potential as candidate genes for studies aimed at developing *B. tryoni* temperature-sensitive GSS. Effective sex separation can be achieved when females homozygous for temperature-sensitive alleles are killed at elevated temperatures, while heterozygous males survive through a functional wild-type allele translocated or transgenically integrated onto the Y chromosome (16). While the *shibire shi^ts1^* G268D allele has previously been shown to have a more severe effect in *B. tryoni* than observed in *D. melanogaster*, other temperature-sensitive *shibire* alleles can be explored.

## Data availability statement

The original contributions presented in the study are included in the article/**Supplementary Material**. Further inquiries can be directed to the corresponding author.

## Ethics statement

The manuscript presents research on animals that do not require ethical approval for their study.

## Author contributions

SB and AC: Conceived the research; SB, AC, and TN: Designed experiments; AC and TN: Performed experiments. TN: Performed data analysis; TN, AC, and SB: Wrote the manuscript. All authors contributed to the article and approved the submitted version.
