# Supplementary material for: Conservation of shibire and RpII215 temperature-sensitive lethal mutations between Drosophila and Bactrocera tryoni
Source: Front Insect Sci. 2024 Mar 4;4:1249103. doi: 10.3389/finsc.2024.1249103 (PMC10926519; doi:10.3389/finsc.2024.1249103)
Supplement: Supplementary file 1 [file DataSheet_1.pdf]

Supplementary Material

Conservation of *shibire* and *RpII215* temperature sensitive lethal mutations between Drosophila and *Bactrocera tryoni*

Thu N. M. Nguyen[1], Amanda Choo[2] and Simon W. Baxter[1*]

First Author, Co-Author, Co-Author

*** Correspondence:** Simon W. Baxter: simon.baxter@unimelb.edu.au

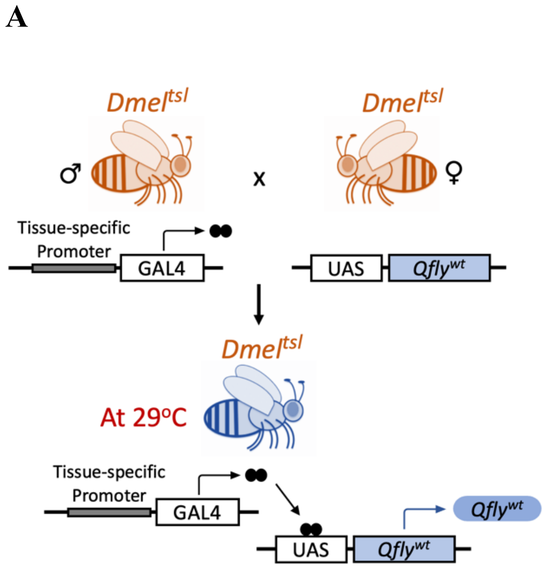

Supplementary Figure S1. Schematic diagram of using *Drosophila melanogaster* GAL4-UAS system to assess whether *Bactrocera tryoni shi* and *RpII215* alleles with equivalent *D. melanogaster* temperature sensitive mutations in *shi* and *RpII215* would render homozygous insects' temperature sensitive. Male flies carrying a tissue-specific GAL4 driver in the *D. melanogaster* temperature sensitive mutant background ($Dmel^{tsl}$) were crossed with female flies carrying UAS-linked *B. tryoni* $Qfly^{wt}$ or $Qfly^{tsl}$ transgene in the $Dmel^{tsl}$ background, producing progeny containing both elements of the system. The GAL4 protein was produced in a tissue-specific manner and will bind to the UAS sites upstream a gene of interest (*B. tryoni* $Qfly^{wt}$ or $Qfly^{tsl}$), hence inducing expression of that gene in a specific tissue. If expression of the *B. tryoni* $Qfly^{wt}$ rescues the lethal phenotype associated with $Dmel^{tsl}$ at the restricted high temperature 29°C and *B. tryoni* $Qfly^{tsl}$ fails to rescue, this indicates that the particular amino acid in the $Qfly^{tsl}$ that differs from $Qfly^{wt}$ is involved in the normal function of the gene at high temperatures.

Supplementary Figure S2. Protein alignment of *Drosophila melanogaster* shibire (shi) "short" isoform and its ortholog on *Bactrocera tryoni.* The *B. tryoni* 'short' isoform was obtained from a gff3 genome annotation file provided by Stuart Gilchrist (Gilchrist et al., 2014), and the 'short' coding sequencing was used generate to *Drosophila* transgenic strains. The other seven *B. tryoni* isoforms (XP_039960167, XP_039960166, XP_039960165, XP_039960164, XP_039960163, XP_039960162 and XP_039960154) were obtained from NCBI, which were derived from a *B. tryoni* chromosome level genome assembly (VWMZ00000000.1). *Drosophila* shi 'short' shares 91% identity with the *B.* tryoni 'short' ortholog. The amino acids that are mutated in the *Drosophila* temperature sensitive shi^{ts1} mutant (G268D) (outlined with a red square) and shi^{ts2} mutant (G141S) (outlined with a blue square) are conserved in *B. tryoni*.

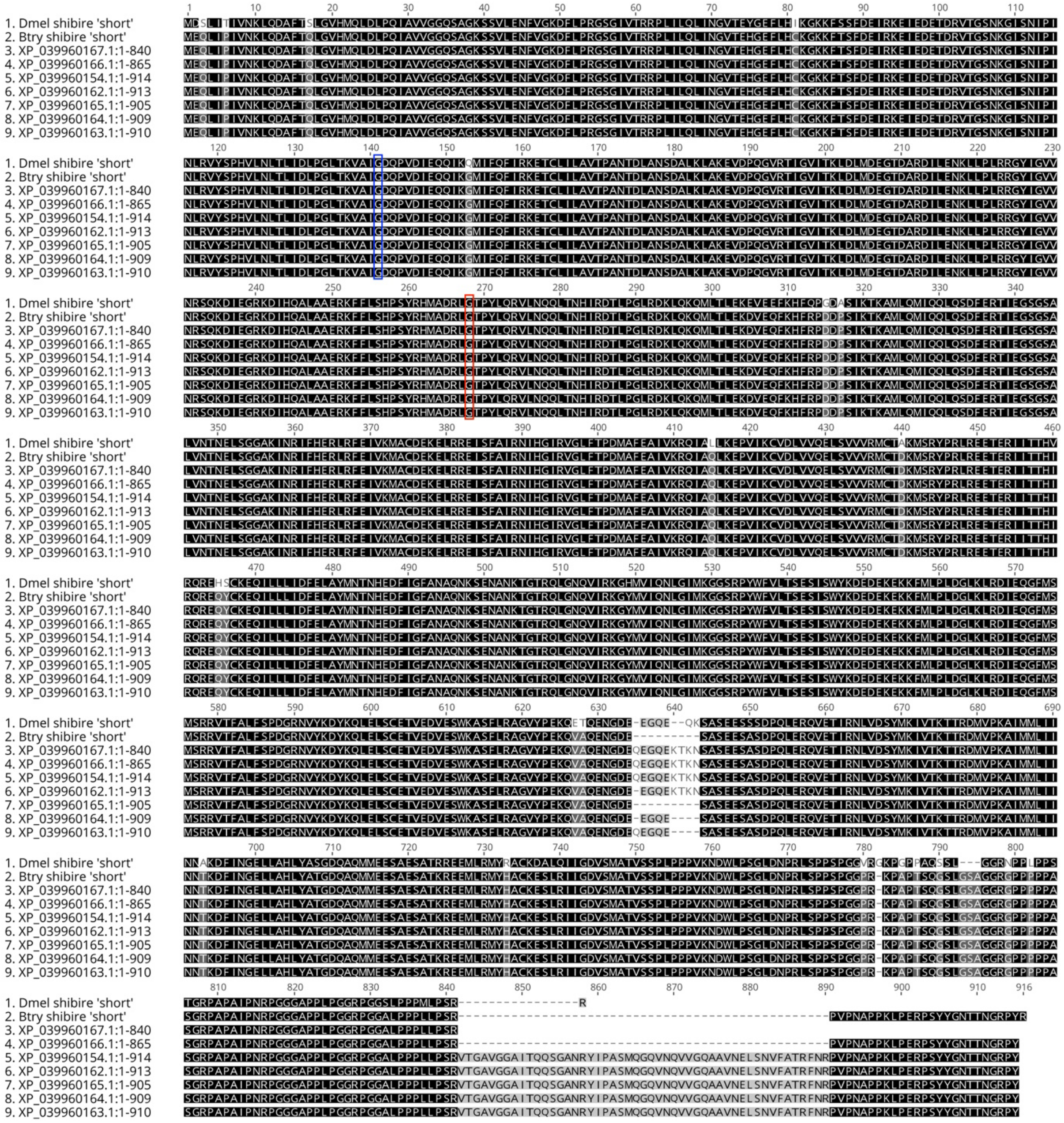

Supplementary Figure S3. Protein alignment of *Drosophila melanogaster* RNA polymerase II 215 (RpII215) isoform and its ortholog on *Bactrocera tryoni.* *Drosophila* RpII215 shares 93% identity with *B.* tryoni ortholog, and the amino acid that is mutated in the *Drosophila* temperature sensitive *RpII215[ts]* mutant (R977C) (outlined with a red square) is conserved in *B. tryoni*.

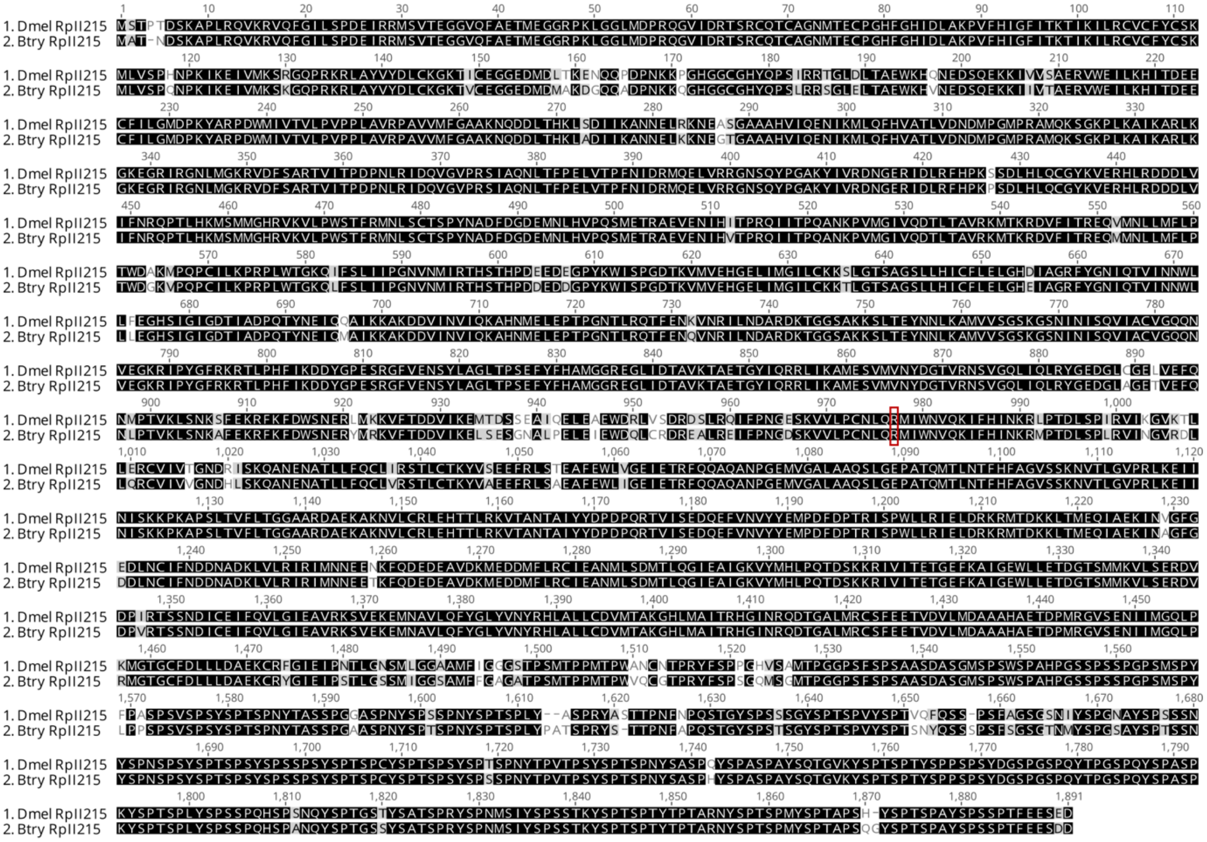

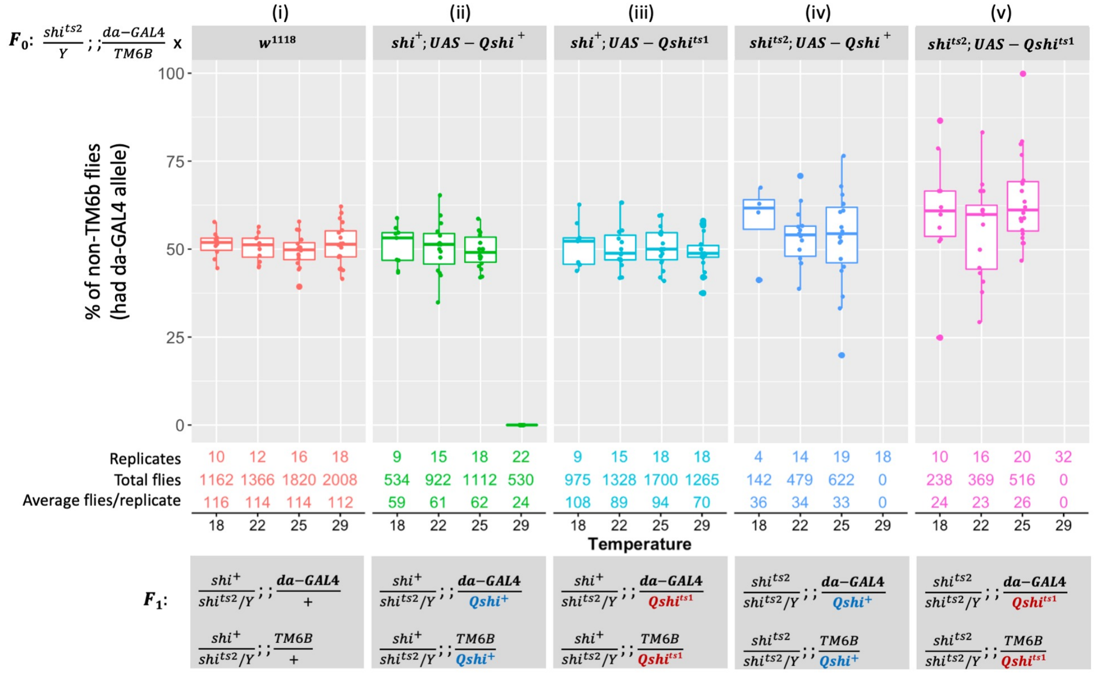

Supplementary Figure S4. Expressing *B. tryoni shi* wild type (*UAS-Qshi*$^{+}$) and mutant (*UAS-Qshi*^{ts1}) transgenes driven by the *shi*^{ts2}*; da-GAL4/TM6B* driver. In each standard food vial, F_0 crosses were set up by placing three males which were hemizygous for the temperature sensitive *shi*^{ts2} mutant allele on the X chromosome, heterozygous for *da-GAL4* and balancer on the 3rd chromosome (*shi*^{ts2}*/Y; +/+; da-GAL4/TM6B*), and five females of either: (i) control wild type (*w*^{1118}), (ii) *Qshi*$^{+}$ in wild type background (*shi*$^{+}$*; UAS-Qshi*$^{+}$), (iii) *Qshi*^{ts1} in wild type background (*shi*$^{+}$*; UAS-Qshi*^{ts1}), (iv) *Qshi*$^{+}$ in the temperature sensitive background (*shi*^{ts2}*; UAS-Qshi*$^{+}$) or (v) *Qshi*^{ts1} in the temperature sensitive background (*shi*^{ts2}*; UAS-Qshi*^{ts1}). F_0 parents were allowed to lay eggs for 24 hours at 25°C, then vial contains eggs were either reared at 18°C, 22°C, 25°C or 29°C. In the F_1, chromosomes are presented in order: sex determination chromosomes; chromosome 2; chromosome 3. F_1 flies with non-TM6B expressed *UAS-Qshi*$^{+}$ or *UAS-Qshi*^{ts1}, while *TM6B* flies do not express those transgenes. The number of replicates and total counted F_1 flies for each cross at each temperature were indicated. Box plots represent the interquartile range, and the median value is indicated. Error bars represent 1.5 times the interquartile range.

Supplementary Table S1. Fly strains used in this study.

**Strain**	**Source/Background**	**Description/Genotype**	**Technique of generation**
w^{1118}	BDSC 51629	white eyes	NA
Canton-S	BDSC 64349	wild type	NA
adar/Fm6	Laboratory stock	Chromosome X balancer	NA
Gla/CyO	Laboratory stock	Chromosome 2 balancer	NA
TM2/TM6B	Laboratory stock	Chromosome 3 balancer	NA
shi-GAL4	BDSC 42738	drive expression ubiquitously (chromosome 2	NA
da-GAL4	BDSC 55850	drive ubiquitous expression at all development stages (chromosome 3)	NA
N-synaptobrevin (*nSyb*)*-Gal4*<br>*nSyb-GAL4*	BDSC 51635	drives expression pan-neuronally at all developmental stages (chromosome 3)	NA
shi^{ts2}	DGRC 106754	heat sensitive (chromosome X)	NA
$Dp(1{:}Y)shi^{+}$	BDSC 4166	wild type shi^{+} *allele* translocated to Y chromosome in wild type shi^{+} background	NA
♀shi^{ts2}/shi^{ts2}<br>♂$shi^{ts2}/Dp(1{:}Y)shi^{+}$	Mixed	females and males are homozygous and hemizygous for shi^{ts2}, males also carry a translocated wild type shi^{+} on the Y chromosome	crossing
shi^{ts2}*; da-GAL4/TM6B*	Mixed	ubiquitous driver under shi^{ts2} mutant background	crossing
shi^{ts2}*; nSyb-GAL4/TM6B*	Mixed	Driving expression in a subset of optic lobe neurons under shi^{ts2} mutant background	crossing
shi^{+}*; UAS-Q*shi^{+}	BestGene	*B. tryoni* wild type *shi* ortholog on 3rd chromosome under wild type shi^{+} background	NA

shi^{+}*; UAS-Qshi*^{ts1}	BestGene	*B. tryoni* temperature sensitive *shi*^{ts1} ortholog on 3rd chromosome under wild type *shi*$^{+}$ background	NA
shi^{ts2}*; UAS-Qshi*$^{+}$	Mixed	*B. tryoni* wild type *shi* ortholog under *shi*^{ts2} mutant background	crossing
shi^{ts2}*; UAS-Qshi*^{ts1}	Mixed	*B. tryoni* temperature sensitive *shi*^{ts1} ortholog under *shi*^{ts2} mutant background	crossing
RpII215^{ts}	BDSC 34755	heat sensitive (chromosome X)	NA
RpII215^{ts}*; da-GAL4/TM6B*	Mixed	heterozygous of ubiquitous driver and *TM6B* dominant marker on 3rd chromosome in *RpII215*^{ts} mutant background	crossing
RpII215^{+}*; UAS-QRpII215*$^{+}$	BestGene	*B. tryoni* wild type *RpII215* ortholog on 2nd chromosome under wild type *RpII215*$^{+}$ background	NA
RpII215^{+}*; UAS-QRpII215*^{ts}	BestGene	*B. tryoni* temperature sensitive *RpII215*^{ts} ortholog on 2nd chromosome under wild type *RpII215*$^{+}$ background	NA
RpII215^{ts}*; UAS-QRpII215*$^{+}$	Mixed	*B. tryoni* wild type *RpII215* ortholog under *RpII215*^{ts} mutant background	crossing
RpII215^{ts}*; UAS-QRpII215*^{ts}	Mixed	*B. tryoni* temperature sensitive *RpII215*^{ts} ortholog under *RpII215*^{ts} mutant background	crossing
UAS-mCD8-GFP	BDSC	express CD8 tagged GFP on the cell membrane under control of UAS	NA

**BDSC = Bloomington Drosophila Stock Centre*

**DGRC = Kyoto Stock Centre*

**NA = not applicable to this strain*

**TM6B: carries dominant balancer Humeral and Tubby. Humeral – adult flies have additional bristles in the humerus; Tubby – larvae, pupae and adults had shorter body compared to wild type*

**♀: female, ♂: male*

**The crossing strategies for strains generated by crossing were illustrated in supplementary materials*

Supplementary Table S2. Strategy for placing *Drosophila melanogaster da-GAL4* driver in the *D. melanogaster shi*^{ts2} mutant background. Chromosomes are presented in order: sex chromosomes; chromosome 2; chromosome 3. '+' refers to wild type and '7' indicates the Y chromosome. The crosses were set up in order from the top to the bottom rows. The progeny used for each cross were coloured in blue. Virgin females were used for each cross. The equivalent crossing strategy were used to place *nSyb-GAL4* driver in the *D. melanogaster shi*^{ts2} mutant background, and *da-GAL4* driver in the *D. melanogaster RpII215*^{ts} mutant background.

$\frac{adar}{Fm6};;\frac{+}{+}$ X $\frac{shi^{ts2}}{7};;\frac{+}{+}$	$\frac{adar}{Fm6};;\frac{+}{+}$ X $\frac{+}{7};;\frac{TM2}{TM6B}$
$\frac{shi^{ts2}}{Fm6};;\frac{+}{+}$ X $\frac{+}{7};;\frac{TM2}{TM6B}$	$\frac{Fm6}{+};;\frac{+}{TM6B}$ X $\frac{+}{7};;\frac{da-GAL4}{da-GAL4}$
$\frac{shi^{ts2}}{Fm6};;\frac{+}{+}$ X $\frac{shi^{ts2}}{7};;\frac{+}{TM6B}$	$\frac{Fm6}{+};;\frac{da-GAL4}{TM6B}$ X $\frac{Fm6}{7};;\frac{da-GAL4}{TM6B}$
$\frac{shi^{ts2}}{Fm6};;\frac{+}{TM6B}$ X $\frac{Fm6}{7};;\frac{da-GAL4}{da-GAL4}$	
$\frac{shi^{ts2}}{Fm6};;\frac{da-GAL4}{TM6B}$ X $\frac{shi^{ts2}}{7};;\frac{da-GAL4}{TM6B}$	
$\frac{shi^{ts2}}{shi^{ts2}};;\frac{da-GAL4}{TM6B}$ X $\frac{shi^{ts2}}{7};;\frac{da-GAL4}{TM6B}$ (Final stock)	

Supplementary Table S3. Strategy for placing *Drosophila melanogaster* mutant *UAS-Qshi^{ts1}* allele in the *D. melanogaster shi^{ts2}* mutant background. Refer to Table S2 for table explanation. The same crossing strategy outlined below was used to place the wild type *UAS-Qshi^{+}* allele in the *D. melanogaster shi^{ts2}* mutant background.

$\frac{adar}{Fm6};;\frac{+}{+}$ x $\frac{shi^{ts2}}{7};;\frac{+}{+}$	$\frac{adar}{Fm6};;\frac{+}{+}$ x $\frac{+}{7};;\frac{TM2}{TM6B}$
$\frac{shi^{ts2}}{Fm6};;\frac{+}{+}$ x $\frac{+}{7};;\frac{TM2}{TM6B}$	$\frac{Fm6}{+};;\frac{+}{TM6B}$ x $\frac{+}{7};;\frac{UAS-Qshi^{ts1}}{UAS-Qshi^{ts1}}$
$\frac{shi^{ts2}}{Fm6};;\frac{+}{+}$ x $\frac{shi^{ts2}}{7};;\frac{+}{TM6B}$	$\frac{Fm6}{+};;\frac{UAS-Qshi^{ts1}}{TM6B}$ x $\frac{Fm6}{7};;\frac{UAS-Qshi^{ts1}}{TM6B}$
$\frac{shi^{ts2}}{Fm6};;\frac{+}{TM6B}$ x $\frac{Fm6}{7};;\frac{UAS-Qshi^{ts1}}{UAS-Qshi^{ts1}}$	
$\frac{shi^{ts2}}{Fm6};;\frac{UAS-Qshi^{ts1}}{TM6B}$ x $\frac{shi^{ts2}}{7};;\frac{UAS-Qshi^{ts1}}{TM6B}$	
$\frac{shi^{ts2}}{shi^{ts2}};;\frac{UAS-Qshi^{ts1}}{UAS-Qshi^{ts1}}$ x $\frac{shi^{ts2}}{7};;\frac{UAS-Qshi^{ts1}}{UAS-Qshi^{ts1}}$ (Final stock)	

Supplementary Table S4. Strategy for placing *Drosophila melanogaster* mutant *UAS-QRpII215*^{ts} allele in the *D. melanogaster RpII215*^{ts} mutant background. The equivalent crossing strategy were used to place wild type *UAS-QRpII215*$^{+}$ allele in the *D. melanogaster RpII215*^{ts} mutant background.

$\frac{adar}{Fm6};\frac{+}{+}$ x $\frac{RpII215^{ts}}{7};\frac{+}{+}$	
$\frac{RpII215^{ts}}{Fm6};\frac{+}{+}$ x $\frac{+}{7};\frac{Gla}{CyO}$	
$\frac{+}{Fm6};\frac{Gla}{+}$ x $\frac{RpII215^{ts}}{7};\frac{+}{CyO}$	$\frac{+}{Fm6};\frac{Gla}{+}$ x $\frac{+}{7};\frac{UAS-QRpII215^{ts}}{CyO}$
$\frac{RpII215^{ts}}{Fm6};\frac{+}{CyO}$ x $\frac{Fm6}{7};\frac{UAS-QRpII215^{ts}}{Gla}$	$\frac{+}{Fm6};\frac{UAS-QRpII215^{ts}}{Gla}$ x $\frac{Fm6}{7};\frac{UAS-QRpII215^{ts}}{Gla}$
$\frac{RpII215^{ts}}{Fm6};\frac{UAS-QRpII215^{ts}}{CyO}$ x $\frac{Fm6}{7};\frac{UAS-QRpII215^{ts}}{Gla}$	
$\frac{RpII215^{ts}}{Fm6};\frac{UAS-QRpII215^{ts}}{UAS-QRpII215^{ts}}$ x $\frac{RpII215^{ts}}{7};\frac{UAS-QRpII215^{ts}}{UAS-QRpII215^{ts}}$	
$\frac{RpII215^{ts}}{RpII215^{ts}};\frac{UAS-QRpII215^{ts}}{UAS-QRpII215^{ts}}$ x $\frac{RpII215^{ts}}{7};\frac{UAS-QRpII215^{ts}}{UAS-QRpII215^{ts}}$ (Final stock)	

Supplementary Table S5. Primers used in this study.

Name	Sequence (5' to 3')	Annealing temperature Tm (ºC)	Product size (bp)	Description
Fwd5917	GGTTACATTGGCGTGGTG	60	814	To amplify and sequence *B. tryoni shi* of the *D. melanogaster* transgenic stocks
Rev6732	CTGAGCATTCGCAAAGCC			
RpII215_Gblock2_F2	GCGATACGGAGAAGATGGTC	60	1461	To amplify and sequence *B. tryoni RpII215* of the *D. melanogaster* transgenic stocks
RpII215_Gblock3_R1	TTCCTGACAGCCTCAA TTCC			
Choo_shits_PCRFwd	GGTTACATTGGCGTGGTG	60	659	To amplify and sequence *D. melanogaster shi*
Choo_shits_PCRRev	CTGAGCATTCGCAAAGCC			
RpII215_F	CGACGGAACAGTGCGTAAC	60	583	To amplify and sequence *D. melanogaster RpII215*
RpII215_R	GACCAACCACTCAAACGCC			

References

Gilchrist, A. S., Shearman, D. C. A., Frommer, M., Raphael, K. A., Deshpande, N. P., Wilkins, M. R., Sherwin, W. B., & Sved, J. A. (2014). The draft genome of the pest tephritid fruit fly Bactrocera tryoni: resources for the genomic analysis of hybridising species. *BMC Genomics, 15*(1), 1153. https://doi.org/10.1186/1471-2164-15-1153
